# Evaluation of Different Doses in Inhaled Therapy: A Comprehensive Analysis

**DOI:** 10.3390/pharmaceutics15092206

**Published:** 2023-08-26

**Authors:** José Luis Lopez-Campos, Rocio Reinoso-Arija, Marta Ferrer Galván, Auxiliadora Romero Falcón, Francisco J. Alvarez-Gutiérrez, Francisco Ortega-Ruiz, Esther Quintana-Gallego

**Affiliations:** 1 Unidad Médico-Quirúrgica de Enfermedades Respiratorias, Instituto de Biomedicina de Sevilla, IBiS, Hospital Universitario Virgen del Rocío, CSIC, Universidad de Sevilla, 41013 Seville, Spain; rocioreari@gmail.com (R.R.-A.); marta.ferrer.sspa@juntadeandalucia.es (M.F.G.); mariaa.romero.sspa@juntadeandalucia.es (A.R.F.); fjavieralvarez2008@gmail.com (F.J.A.-G.); francisco.ortega.sspa@juntadeandalucia.es (F.O.-R.); esther.quintana@telefonica.net (E.Q.-G.); 2 Centro de Investigación Biomédica en Red de Enfermedades Respiratorias (CIBERES), Instituto de Salud Carlos III, 28029 Madrid, Spain

**Keywords:** inhaled drugs, COPD, asthma, dose, inhalers

## Abstract

Background. Currently, there is a considerable degree of confusion over the dosage of inhaled medications. Here, we carried out a review of all the doses used for the devices used in inhalation therapy. Methods. We first performed a systematic search of the different inhalation devices included on the July 2023 Spanish Ministry of Health Billing List. We then consulted the Spanish Agency for Medicines and Health Products to find the updated official label and to obtain the information on the exact composition. Results. We identified 90 unique products, of which 22 were long-acting bronchodilators (and combinations thereof) and 68 were products containing inhaled corticosteroids (ICS). Overall, 10 products with bronchodilators and 40 with ICS were marketed with the metered dose, while 11 with bronchodilators and 28 with ICS were marketed with the delivered dose. In addition, in some bronchodilators, the drug was referred to as a type of salt, whereas in others the information referred to the drug itself. Conclusions. Our data show that for each inhaled drug there may be up to four different doses and that the marketed name may refer to any of these. Clinicians must be aware of these different dosages when prescribing inhaled medications.

## 1. Introduction

Due to its considerable advantages, inhaled therapy continues to be the mainstay in the pharmacological treatment of airway diseases [1,2,3,4]. Among its advantages are the low dose, direct deposition in the target organ, and low bioavailability. However, one of the major limitations is how to ensure that the patient uses a suitable inhalation technique [5]; more recently, the impact of inhalers on climate change has also been the subject of debate [6]. In order to overcome some of the technical limitations that inhalers can have, different manufacturers have been innovating new ways of administering drugs via the inhaled route by the development of new inhalation devices [7]. Initially, having different inhalation devices can be an advantage, since this allows the clinician to adapt to the needs of each patient, choosing the best device in each case to administer the same dose of the drug. However, in recent years, this has led to changes in the doses of inhaled drugs depending on the device used [8], with the added problem that inhalation devices have different types of doses. On the one hand, the so-called metered dose refers to the dose of medication within the device available to deliver with each puff; on the other hand, the delivered dose refers to the dose that actually comes out when a dose is taken. Interestingly, among the different marketed presentations of inhaled drugs, some refer to the metered dose and others to the delivered dose.

This situation has led to considerable confusion among clinicians about the administered dose when it comes to prescribing an inhaler. In the present study, we carried out a review of all the doses of inhalation devices available in Spain in order to clarify the dosage of inhaled drugs, as well as to discuss the changes that have been made in the different presentations. The results will help clinicians understand better the actual doses of drugs being administered to patients.

## 2. Materials and Methods

The present study is a cross-sectional analysis of the current situation of inhaled therapy in Spain and is based on a systematic search of the different inhalation devices available in the country in July 2023. To include all the inhalation devices available, without exceptions, we performed a search on the Spanish Ministry of Health Billing List (https://www.sanidad.gob.es/profesionales/nomenclator.do (accessed on 22 August 2023)). This billing list provides information on all the products included within the National Health System pharmaceutical provision that are available in pharmacies. It is freely accessible to the public and provides basic information for healthcare professionals on each drug, with information on its marketing, availability, characteristics, and current price. For the present study, we used this database exclusively to obtain the list of inhalers available when the study was carried out. The search was performed for each of the active principles available for inhalers in Spain with the three different families of molecules: long-acting ß agonists (LABA), long-acting muscarinic antagonists (LAMA), and inhaled corticosteroids (ICS). We also carried out a secondary search on the same database using the names of the inhalers. For simplicity, trade names representing licenses of the same molecule or combination of molecules from the original laboratory were considered as a single option and were referred to in the results with the original commercial brand name used in Spain. Each inhaled drug or drug combination was then identified by the original trade name and its accompanying inhalation device, along with the dose quantity appearing on the publicly available commercial package.

After obtaining the list of inhaled drugs or drug combinations, we consulted the Spanish Agency for Medicines and Health Products (https://www.aemps.gob.es/ (accessed on 22 August 2023)), a branch of the Ministry of Health, to find the most recent technical data sheet for each one, consulting specifically Section 2 of the document to obtain information on the exact composition of each product. These showed us the molecules they contained for therapeutic purposes, excluding excipients, and were expressed in µg per dose, with two types of dose: the metered dose, which is the dose contained in the device available to be inhaled, and the delivered dose, which is the dose that actually comes out of the inhaler and reaches the patient’s lungs during the inhalation procedure. Additionally, for each type of dose (metered and delivered), two other types of doses are also mentioned when available: the dose that refers to the molecule with therapeutic action combined with a transporter in salt form, and the dose that refers to the amount of the pure drug. For example, in the case of tiotropium bromide, the amount of µg per dose of both tiotropium bromide and tiotropium alone was specified. In this way, we could have up to four different doses for each molecule. The results showed both the drugs or their combinations of long-acting bronchodilators (LABA and/or LAMA) and the ICS with their double and triple combinations. 

## 3. Results

During the search, we identified 90 unique products (some of which had more than one commercial brand name) available in Spain for inhalation to treat airway diseases. The list of long-acting bronchodilators and their combinations was composed of 22 unique products (Table 1). As can be seen, 10 products are marketed with the metered dose, while 11 are marketed with the delivered dose. Interestingly, the information for the combination of aclidinium with formoterol contains the metered dose for formoterol and the delivered dose for aclidinium (Table 1d). In addition, with LAMAs, the label on the marketed package usually corresponds to the pure drug, while LABAs usually refer to the combined drug in salt form. The combination of tiotropium and olodaterol has a higher dose of tiotropium when given as single treatment than when combined with olodaterol (Table 1b); also, in the case of the combination of indacaterol with glycopyrronium (Table 1c), a slightly lower dose of glycopyrronium and a much lower dose of indacaterol are indicated than the dose used in monotherapy.

The search for products containing ICS resulted in a list of 68 different products (Table 2). Similar to findings for bronchodilators, 40 products are marketed with the metered dose, while 28 are marketed with the delivered dose. The extra-fine formulation of beclomethasone (Table 2b) is marketed with lower doses than the original fine particle presentation (Table 2a). Standard doses of budesonide are presentations of a 100, 200, and 400 µg metered dose, with the exception of the Breezhaler presentation (Table 2c), where the metered dose is higher to achieve a delivered dose of 200 and 400 µg. These presentations in Breezhaler therefore have a higher content of budesonide. Interestingly, the triple combination of budesonide, formoterol, and glycopyrronium uses a lower dose for glycopyrronium, similar to the one approved in the US.

The combinations of fluticasone propionate + salmeterol (Table 2e) and fluticasone + formoterol (Table 2f) follow a very similar dose content in the metered dose. All these inhalers are marketed with the metered dose, except for the Spiromax presentation. This inhaler contains a delivered dose of 12.75 µg of salmeterol xinafoate and 100/202 µg of fluticasone propionate. Of note, the marketed information for combinations with fluticasone furoate refers to the delivered dose.

Combinations with mometasone are marketed in a presentation with a Breezhaler device, where all three presentations of the LABA-ICS combination have progressively higher doses for the stepped treatment of bronchial asthma. Interestingly, the triple combination, which is intended to treat severe asthma, specifies a medium dose of ICS (Table 2h).

## 4. Discussion

This article reviews the dosages of the different inhalers marketed in Spain for the treatment of airway diseases, mainly asthma and chronic obstructive pulmonary disease. Our analysis shows that the dosage that appears in the marketed information for each drug is confusing and can be misleading, referring indistinctly to the measured dose or the delivered dose, and to the combined drug in salt form or to the pure compound. This situation is complicated further in the presentations of combined drugs, in which each drug in the combination can follow a different rule. Consequently, choosing the dosage of inhaled drugs for airway diseases presents a real challenge for clinicians.

The treatment of airway diseases with inhalation devices has a number of major advantages over the oral route [9]. The doses used are lower, the onset of action is faster, the distribution is more limited to the target organ, with less bioavailability, and the potential adverse effects are mainly local. Consequently, inhaled therapy represents an unparalleled opportunity for the treatment of airway diseases. However, despite its advantages, inhaled therapy also has its challenges. Not only does the correct inhalation technique probably represent the main barrier to be overcome, but, as seen here, the marketed dosages of the inhaled drugs can also be extremely confusing, and clinicians must take great care what dosages they prescribe.

The data presented in this paper highlight the considerable confusion that can exist when choosing doses for inhaled therapy. Some of the combinations marketed in Spain deserve comment. For instance, the reformulation of beclomethasone dipropionate to change the propellant to chlorofluorocarbon-free hydrofluoroalkane-134a provided an opportunity to produce a formulated solution that provides a higher total mass of fine drug particles [10]. While the old formulation of beclomethasone carried two chlorofluorocarbon propellants and a surfactant which produced a suspension, the new beclomethasone formulated with the propellant hydrofluoroalkane is a solution without added surfactant. As a consequence, this new formulation releases an aerosol with particles with a mass mean aerodynamic diameter of 1.1 µm, whereas the old suspension released particles with a mass mean aerodynamic diameter of 3.5–4.0 µm [10]. This has led to a reduction in the total dose of the ICS, compared to the fine particle presentation (Table 2b). Interestingly, the lower drug dose shown here for ICS does not appear to be the same for bronchodilators. In fact, the combinations of bronchodilators with beclomethasone indicate a lower dose for ICS, but the same dose for long-acting bronchodilators (Table 2b). Of note, the use of next-generation propellants with a significantly lower environmental impact are under development, with new candidate options [11]. It is therefore possible that these new propellants will impact the final dose for future inhalers.

The combinations of formoterol with budesonide (Table 2c) present a similar relationship between metered-dose delivered and dose. The doses of the combination of budesonide with formoterol are expressed in the packages in terms of the delivered dose, where the combination 400/12 µg metered dose corresponds to 320/9 µg delivered dose, and 200/6 µg metered dose corresponds to 160/4.5 µg delivered dose. We should bear in mind that the approved treatment dose for formoterol is 12 µg every 12 h for the metered dose. Consequently, it should be inhaled once or twice every 12 h, depending on the presentation of the bronchodilator. However, the presentation of budesonide + formoterol in the Easyhaler device provides a notable exception, where the label specifies that the delivered dose is very similar to the metered dose.

The dosage of glycopyrronium also deserves a comment. Glycopyrronium bromide is available as a stand-alone drug in many forms: in the Breezhaler device, combined with indacaterol in Breezhaler, in triple therapy with beclomethasone and formoterol for both pressurized metered-dose and Nexthaler devices, in triple therapy with budesonide and formoterol for a pressurized metered-dose inhaler, and in triple therapy with mometasone and indacaterol in the Breezhaler device (Table 2). In each of these combinations, the specified dosage is different. The approved dose for the use of glycopyrronium in Europe is formerly 50 µg per day delivered dose [12], which corresponds to 63 µg of glycopyrronium bromide metered dose. However, in the USA, its use has been approved with a metered dose of 15.6 µg of glycopyrronium bromide, administered twice daily, resulting in a daily dose of 31.2 µg of glycopyrronium bromide per metered dose per day [13,14]. Interestingly, the dose of glycopyrronium used in combinations in Europe corresponds to the European dose, either administered once a day or divided into two doses a day, with the exception of the triple combination with budesonide and formoterol, which has a similar dose to the American one. In this latter combination, a lower dose is specified for indacaterol than when it is administered as a monotherapy. This effect has been associated with the use of magnesium stearate as an excipient, which is not present when indacaterol is administered alone and is present with glycopyrronium in isolated treatment. The magnesium stearate excipient has been described as a ‘Force Control Agent’ which increases the dispersibility of drug particles, thereby enabling optimal aerosolization [15,16]

There are two different salmeterol-fluticasone Spiromax presentations, one with the standard dose of 50/500 µg and another that contains a delivered dose of 12.75 µg of Salmeterol xinafoate and 100/202 µg of fluticasone propionate (Table 2e), which is only half the dose of the other presentations with this salmeterol + fluticasone combination. These results are based on dose-ranging and pharmacokinetic studies that compare the Spiromax presentation with the traditional 50/500 µg or 50/250 µg presentations in asthma patients [17,18,19], and it opens up considerable debate about how the optimal doses in inhaled therapy are calculated.

Finally, mometasone furoate is designated a different dosage depending on whether it is used with a Twisthaler or a Breezhaler, with a considerably lower dosage for the latter. This lower dose resulted from a randomized, 4-week clinical study in asthma patients comparing the different strengths in both inhalers [20]. The study showed comparable improvements from baseline in trough FEV1 for the corresponding ICS doses. Interestingly, the triple combination of mometasone, indacaterol, and glycopyrronium in the Breezhaler presentation, intended to treat severe asthma, has a lower dose of ICS compared with the same LABA/ICS combination (Table 2h). This results from bridging studies, which determined that mometasone furoate 160 µg in the triple combination formulation provided comparable ICS efficacy to mometasone furoate 160 µg and 320 µg in the LABA/ICS formulation [21]. In the triple co-formulation, an increase in the mometasone furoate fine particle mass was observed compared to the corresponding LABA/ICS due to pharmaceutical interaction with glycopyrronium. A dose adjustment was therefore carried out to reduce the nominal doses of mometasone furoate and equal 160 µg strength to 320 µg.

One of the most worrying consequences of these different dosages may be related to the ICS dose and asthma control. The different dosages of the inhalers are of special importance in the stepped treatment of bronchial asthma. According to current recommendations, the severity of asthma is established according to the dose of ICS needed to achieve control of the disease. Consequently, to manage asthma successfully, it is essential to be clear about what amount of drug corresponds to a low, medium, or high dose of ICS. However, our results indicate that the dose can be confusing, depending on whether it is considered a metered or delivered dose. Here, the international GINA guidelines establish the different strengths of ICS according to the metered dose [1], while the Spanish guidelines mixes metered doses with delivered doses in their recommendations [2]. In addition, our results show that ICS doses change when ICS is administered alone or accompanied by bronchodilators (e.g., mometasone and its combinations, Table 2h). Of note, in the case of Spiromax, this dose changed, even with different commercial products that use the same inhalation device (Table 2e). These examples undoubtedly contribute considerably to establishing the correct dosage intensity for ICS, which is crucial in the treatment of asthma. In this context, it is important to bear in mind that the delivered dose is obtained through an in vitro assay, producing an Inhalation at a known flow rate that informs about both the internal resistance of the inhaler and the delivered dose [22]. Interestingly, the flow rate used with the inhaler to carry out this test is not standardized between the different devices, and ranges between 30 and 90 L/min, despite the fact that we have known for a long time that flow rate is a factor that decisively influences the behavior of the inhaler [23,24]. Consequently, the emitted dose may change depending on the conditions under which this in vitro study is carried out. Therefore, clinicians should be particularly careful with the real metered dose they are prescribing to their patients, since delivered doses are based on estimations which may vary with the inhalation technique, the severity of the disease and the characteristics of each patient’s airway.

The present study provides a thorough review of the inhalation devices available in Spain. In this context, it is necessary to take into account certain methodological limitations. The information analyzed has been obtained from the technical data information sheets for the products available in Spain, and not with data obtained directly from patients or from an ad hoc in vitro study. Secondly, the data refer to products marketed in Spain, and other countries may specify other doses for these products or for other inhalation devices. Finally, we have only analyzed inhaled long-acting bronchodilators and ICS. Other inhaled drugs such as antibiotics [25] and, in the near future, other potential drugs that will be administered in inhaled form [26] have not been considered in this article, nor has nebulized therapy [27].

In conclusion, the doses of inhaled drugs can be expressed in a number of ways, which can lead to some confusion. These dosages depend on three factors: the inhalation device, whether it is a metered or delivered dose, and whether we consider the combined drug in salt form or in isolation. In this way, there can be up to four values to refer to the same dose for each inhaler, and the information that appears on the packaging of each product can refer to any of them. Therefore, to avoid this confusion, there should be an agreed strategy among all manufacturers on the best way to indicate the doses of inhalation devices. The clinician must keep these different dosages in mind to be clear at all times the amount of drug that is being prescribed, so as to guarantee maximum effectiveness while ensuring an adequate safety profile.

## Figures and Tables

**Table 1:** List of long-acting bronchodilators and their combinations available showing their metered and delivered doses.

(a) Salmeterol				
Product name when first marketed in Spain *	Metered dose ^†^		Delivered dose ^†^	
	–	Salmeterol xinafoate	–	Salmeterol xinafoate
Serevent MDI 25	–	25	–	NA
Serevent Accuhaler 50	–	50	–	NA
(b) Tiotropium-olodaterol				
Product name when first marketed in Spain *	Metered dose ^†^		Delivered dose ^†^	
	Tiotropium bromide monohydrate	Olodaterol hydrochloride	Tiotropium bromide monohydrate	Olodaterol hydrochloride
Spiriva Handihaler 18	22.5 (18)	–	? (10)	–
Braltus Zonda 10	16 (13)	–	? (10)	–
Tavulus Inhalator 18	21.7 (18)	–	? (10)	–
Spiriva Respimat 2.5	NA	–	3.124 (2.5)	–
Striverdi Respimat 2.5	–	NA	–	2.5
Spiolto Respimat 2.5/2.5	NA	NA	2.5	2.5
(c) Glycopyrronium-indacaterol				
Product name when first marketed in Spain *	Metered dose ^†^		Delivered dose ^†^	
	Glycopyrronium bromide	Indacaterol maleate	Glycopyrronium bromide	Indacaterol maleate
Onbrez Breezhaler 150	–	? (150)	–	? (120)
Onbrez Breezhaler 300	–	? (300)	–	? (240)
Seebri Breezhaler 44	63 (50)	–	55 (44)	–
Ultibro Breezhaler 85/43	63 (50)	143 (110)	54 (43)	110 (85)
(d) Aclidinium-formoterol				
Product name when first marketed in Spain *	Metered dose ^†^		Delivered dose ^†^	
	Aclidinium bromide	Formoterol fumarate dihydrate	Aclidinium bromide	Formoterol fumarate dihydrate
Foradil Areolizer 12	–	12	–	?
Foradil Neo MDI 12	–	12	–	10.1
Formatris Novolizer 12	–	12	–	10.2 (8.36)
Formatris Novolizer 6	–	6		5.1 (4.18)
Oxis Turbuhaler 9	–	12	–	9
Oxis Turbuhaler 4.5	–	6	–	4.5
Eklira Genuair 322	400 (343)	–	375 (322)	–
Duaklir Genuair 340/12	400 (343)	12	396 (340)	11.8
(e) Umeclidinium-vilanterol				
Product name when first marketed in Spain *	Metered dose ^†^		Delivered dose ^†^	
	Umeclidinium bromide	Vilanterol trifenatate	Umeclidinium bromide	Vilanterol trifenatate
Incruse Ellipta 55	74.2 (62.5)	–	65 (55)	–
Anoro Ellipta 55/22	74.2 (62.5)	25	65 (55)	22

* Name refers to the first commercial brand name available in Spain. Additionally, some products may have other commercial brand names. ^†^ Dose expressed in µg per puff of the combined drug in salt form. In brackets, the corresponding dose of the drug alone, when available. NA: not available for the official label or the product. ?: unknown. MDI: Metered-dose inhaler. The dose that appears on the marketed package is indicated in gray.

**Table 2:** List of inhaled corticosteroids and their combinations available showing their metered and delivered doses.

(a) Beclomethasone						
Product name when first marketed in Spain *	Metered dose ^†^			Delivered dose ^†^		
	Beclomethasone dipropionate	–	–	Beclomethasone dipropionate	–	–
Beclo-Asma MDI 50	50	–	–	NA	–	–
Beclo-Asma MDI 100	100	–	–	NA	–	–
Becloforte MDI 250	250	–	–	NA	–	–
Becotide MDI 50	50	–	–	NA	–	–
Soprobec MDI 50	50	–	–	NA	–	–
Soprobec MDI 100	100	–	–	NA	–	–
Soprobec MDI 200	200	–	–	NA	–	–
Soprobec MDI 250	250	–	–	NA	–	–
(b) Beclomethasone extra fine						
Product name when first marketed in Spain *	Metered dose ^†^			Delivered dose ^†^		
	Beclomethasone dipropionate	Formoterol fumarate dihydrate	Glycopyrronium bromide	Beclomethasone dipropionate	Formoterol fumarate dihydrate	Glycopyrronium bromide
Foster MDI 100/6	100	6	–	84.6	5.0	–
Foster MDI 200/6	200	6	–	177.7	5.1	–
Foster Nexthaler 100/6	100	6	–	81.9	5.0	–
Foster Nexthaler 200/6	200	6	–	158.8	4.9	–
Trimbow MDI 87/5/9	100	6	12.5 (10)	87	5.0	11 (9)
Trimbow MDI 172/5/9	200	6	12.5 (10)	172	5.0	11 (9)
Trimbow Nexthaler 88/5/9	100	6	12.5 (10)	88	5.0	11 (9)
(c) Budesonide						
Product name when first marketed in Spain *	Metered dose ^†^			Delivered dose ^†^		
	Budesonide	Formoterol fumarate dihydrate	Glycopyrronium bromide	Budesonide	Formoterol fumarate dihydrate	Glycopyrronium bromide
Pulmicort Turbuhaler 100	100	–	–	?	–	–
Pulmicort Turbuhaler 200	200	–	–	?	–	–
Pulmicort Turbuhaler 400	400	–	–	?	–	–
Budesonida Easyhaler 100	100	–	–	100	–	–
Budesonida Easyhaler 200	200	–	–	200	–	–
Budesonida Easyhaler 400	400	–	–	400	–	–
Miflonide Breezhaler 200	230	–	–	200	–	–
Miflonide Breezhaler 400	460	–	–	400	–	–
Symbicort Turbuhaler 80/4.5	100	6	–	80	4.5	–
Symbicort Turbuhaler 160/4.5	200	6	–	160	4.5	–
Symbicort Turbuhaler 320/9	400	12	–	320	9	–
Symbicort MDI 80/2.25	100	3	–	80	2.25	–
Symbicort MDI 160/4.5	200	6	–	160	4.5	–
Symbicort MDI 320/9	400	12	–	320	9	–
Duoresp Spiromax 160/4.5	200	6	–	160	4.5	–
Duoresp Spiromax 320/9	400	12	–	320	9	–
Bufomix Easyhaler 160/4.5	160	4.5	–	160	4.5	–
Bufomix Easyhaler 320/9	320	9	–	320	9	–
Trixeo Aerosphere 5/7.2/160	170	5.3	9.6 (7.7)	160	5	9 (7.2)
(d) Ciclesonide						
Product name when first marketed in Spain *	Metered dose ^†^			Delivered dose ^†^		
	Ciclesonide	–	–	Ciclesonide	–	–
Alvesco MDI 160	200	–	–	160	–	–
(e) Fluticasone propionate + salmeterol						
Product name as first marketed in Spain *	Metered dose ^†^			Delivered dose ^†^		
	Fluticasone propionate	Salmeterol xinafoate	–	Fluticasone propionate	Salmeterol xinafoate	–
Flixotide MDI 50	50	–	–	NA	–	–
Flixotide MDI 125	125	–	–	NA	–	–
Flixotide MDI 250	250	–	–	NA	–	–
Flixotide Accuhaler 100	100	–	–	NA	–	–
Flixotide Accuhaler 500	500	–	–	NA	–	–
Seretide MDI 25/50	50	25	–	44	21	–
Seretide MDI 25/125	125	25	–	110	21	–
Seretide MDI 25/250	250	25	–	220	21	–
Seretide Accuhaler 50/100	100	50	–	92	47	–
Seretide Accuhaler 50/250	250	50	–	231	47	–
Seretide Accuhaler 50/500	500	50	–	460	47	–
Flusamix Easyhaler 50/500	500	50	–	476	48	–
Aerivio Spiromax 50/500	500	50	–	465	45	–
Airflusal Forspiro 50/250	250	50	–	233	45	–
Airflusal Forspiro 50/500	500	50	–	465	45	–
Inhalok Airmaster 50/250	250	50	–	229	45	–
Inhalok Airmaster 50/500	500	50	–	432	45	–
Seffalair Spiromax 12.75/100	113	14	–	100	12.75	–
Seffalair Spiromax 12.75/202	232	14	–	202	12.75	–
(f) Fluticasone propionate + formoterol						
Product name when first marketed in Spain *	Metered dose ^†^			Delivered dose ^†^		
	Fluticasone propionate	Formoterol fumarate dihydrate	–	Fluticasone propionate	Formoterol fumarate dihydrate	–
Flutiform MDI 5/50	50	5	–	46	4.5	–
Flutiform MDI 5/125	125	5	–	115	4.5	–
Flutiform MDI 10/250	250	10	–	230	9.0	–
Flutiform K-haler 5/50	50	5	–	46	4.5	–
Flutiform K-haler 5/125	125	5	–	115	4.5	–
(g) Fluticasone furoate						
Product name when first marketed in Spain *	Metered dose ^†^			Delivered dose ^†^		
	Fluticasone furoate	Vilanterol trifenatate	Umeclidinium bromide	Fluticasone furoate	Vilanterol trifenatate	Umeclidinium bromide
Relvar Ellipta 92/22	100	25	–	92	22	–
Relvar Ellipta 184/22	200	25	–	184	22	–
Trelegy Ellipta 92/55/22	100	25	74.2 (62.5)	92	22	65 (55)
(h) Mometasone						
Product name when first marketed in Spain *	Metered dose ^†^			Delivered dose ^†^		
	Mometasone furoate	Indacaterol acetate	Glycopyrronium bromide	Mometasone furoate	Indacaterol acetate	Glycopyrronium bromide
Asmanex Twisthaler 200	220	–	–	200	–	–
Asmanex Twisthaler 400	NA	–	–	400	–	–
Atectura Breezhaler 125/62.5	80	150	–	62.5	125	–
Atectura Breezhaler 125/125.7	160	150	–	125.7	125	–
Atectura Breezhaler 125/260	320	150	–	260	125	–
Enerzair Breezhaler 114/46/136	160	150	63 (50)	136	114	58 (46)

* Name refers to first commercial brand name available in Spain. Additionally, some products may have other commercial brand names. ^†^ Dose expressed in µg per puff of the combined drug in the form of salt. In brackets, the corresponding dose of the drug alone, when available. NA: not available for the official label or the product. ?: unknown. MDI: Metered-dose inhaler. The dose that appears on the marketed package is indicated in gray.

## Data Availability

The authors confirm that the data supporting the findings of this study are available within the article.
